# Revision of medicine prices and sales price evolution in Moroccan pharmacies

**DOI:** 10.11604/pamj.2025.51.28.45569

**Published:** 2025-06-04

**Authors:** Fatima Zahra Bimegdi, Abdelmajid Belaiche, Samir Ahid

**Affiliations:** 1 Research team of Pharmacoeconomics and Pharmacoepidemiology, Laboratory of Pharmacology and Toxicology, Faculty of Medicine and Pharmacy, Mohammed V University of Rabat, Impasse Souissi, Rabat, Morocco,; 2 Euromed Research Center, Euromed Faculty of Pharmacy, Euromed University of Fes, National Road Fes Meknes, Fes, Morocco

**Keywords:** Medicines, price regulation, price revisions, price decreases, pharmacies, Morocco

## Abstract

**Introduction:**

Morocco adopted a new medicines pricing policy in 2013 that introduced price revisions following the implementation of Decree No. 2-13-852. This study aims to describe the evolution in medicine price decreases in Moroccan pharmacies following the price revisions published by the Ministry of Health.

**Methods:**

as part of a retrospective descriptive study, all the price revision lists over five years (2014-2019) were crossed with the databases of sales of medicines sold in pharmacies.

**Results:**

price decrease affected 1704 medicines, 54% of which were originator drugs. All therapeutic classes were involved, 22.7% were anti-infective, and 18.5% were for the cardiovascular system. Medicines with a public selling price between 5 and 10 US dollars (25.4%) were the most affected by the drop, even if it has concerned all price ranges. 22.8% of medicines decreased by less than or equal to 0.1 US dollar, and 28.7% recorded decreases of less than or equal to 1% of the public selling price. Five medicines (0.3%) have been reduced by over 70%, with 73.2% as a maximum drop percentage.

**Conclusion:**

following the implementation of Decree no. 2-13-852, a series of medicine price revisions was adopted. All price ranges and therapeutic classes were affected by the price decrease, with several variations. However, complementary policies are still needed to improve access to medicines.

## Introduction

Medicines allow prevention and treatment of disease while safeguarding public health, to reduce both morbidity and mortality rates. The price at which medicines are sold is an important factor for accessibility [1]. Affordability is one of the major health challenges facing all countries, especially as many prices exceed affordability thresholds [2]. The World Health Organization indicates that between 20% and 60% of healthcare expenditure in developing countries is linked to the purchase of medicines [3]. Expenditure on medicines can be variable and depends on the methods used by different governments to set drug prices, even when countries have similar economic levels [4]. The price of this product is set according to the national regulations through health policy in general and pharmaceutical sector policy in particular [5] since there is no single system adopted by all countries [6]. However, in recent years, there have been several debates about the high prices of medicines [7]. In Morocco, the national pharmaceutical policy represents the strategic vision of the Ministry of Health (MH) for the pharmaceutical sector. One of its objectives is to improve access to medicines for citizens. Regulating the price of medicines is one of its pillars [8]. It represents the set of principles necessary for the proper management of prices adopted by the competent bodies and aims to guarantee both fair and affordable access to medicines [5] while ensuring that health insurance funds are balanced. Despite the fact that Morocco has a strong pharmaceutical industry which meets the national need for medicines by manufacturing nearly 60% of requirements locally and importing 40%, with the aim of achieving self-sufficiency and substituting manufacturing for imports [9,10], it is still faced with limited purchasing power and inadequate health insurance cover [11].

For more than two decades, the issues of access to medicines and their prices have been at the heart of the national conversation between stakeholders in the health and pharmaceutical sectors [12]. According to the MH, households contribute 50% of health funding [13] while medicines account for 22% of health expenditure among Moroccans [14]. In response to the various debates on the price of medicines and their high cost in Morocco, in which several components of the health sector have taken part [15], the MH has unveiled the Decree revising the regulations for setting the price of medicines, which includes a reduction in the price of numerous medicines. Prior to its revision, pricing regulation in Morocco dated from 1969, with separate provisions for locally manufactured and imported pharmaceutical products, which no longer met the country's economic parameters [16]. A different method of fixing medicine prices was adopted at the end of 2013. Decree N. 2-13-852 relating to the conditions and procedures for setting the Public Selling Price (PSP) of medicines manufactured locally or imported and having obtained a marketing authorization sets out new regulatory measures. For fixing the price of a medicine, three main factors are taken into account: the Manufacturer Price Excluding Taxes (MPET), the distribution margins of the pharmaceutical wholesaler-distributor and the dispensing pharmacist, and value-added tax (VAT) where applicable.

The introduction of the Benchmark has been adopted and the MPET of a locally manufactured or imported originator drug must be the lowest of the MPET of the same drug in the six countries selected, i.e. Belgium, France, Portugal, Saudi Arabia, Spain and Turkey, and in the country of origin if it is not one of these countries. The distribution margins of the pharmaceutical wholesaler-distributor, the dispensing pharmacist and VAT when applicable, are added to the MPET [17]. According to the provisions of this Decree, downward revisions of the PSP of any medicinal product may be applied in three situations: at the request of the industrial pharmaceutical establishment, if it appears to the MH that the MPET applied in the Benchmark countries and in the country of origin - if different from the latter - have fallen by more than 10%, or if the medicinal product concerned is exempt from VAT. In addition, the dispensing pharmacist's margin, as stipulated in this law, is no longer fixed, but variable and inversely proportional to the price, whereas above a net PSP of 58.8 US dollars (USD), the margin is replaced by a fixed fee [17]. From April 2014, following the implementation of this pricing system, the MH published several lists of revised drug prices in official bulletins (OB). These lists set out the prices before and after the revision, applied separately in pharmacies and in hospitals. To date, to our knowledge, no study has been carried out on the prices of medicines appearing on the medicine sales databases and all the price revision lists for medicines sold in Moroccan pharmacies between 2014 and 2019. The aim of this study is to provide an overview of changes in the prices of medicines sold in pharmacies affected by the price decrease.

## Methods

**Study design and setting:** the study design was a retrospective descriptive study of the evolution of price decreases for medicines sold in pharmacies in Morocco after the MH price revisions. The latter have been applied since the publication of the decree n° 2-13-852 in December 2013. We chose a period of five years (2014-2019) after the implementation of this decree to have a sufficient transition period for the application of price revisions.

**Data collection:** we collected all lists of medicine price revisions published by the MH in the OB at the General Secretariat of the Government from the implementation of the decree in 2014 until September 2019. Their total number was 28 lists and included the commercial name of the medicine and its presentation, the PSP before revision, and the PSP after revision. They were crossed with the IQVIA (Ex IMS Health) Morocco medicine sales databases, which corresponded to drug sales by pharmaceutical laboratories and wholesale distributors to pharmacies, representing 90% of the private pharmaceutical market. The sales databases included 4266 medicines that were marketed in Morocco through pharmacies for the study period. Each medicine was completed with its price before and after revision, as shown on the OB. In order to meet the objective of our study, which concerns the evolution of medicine price decreases following revisions, price difference before and after the revision allowed us to identify a sample of 1704 medicines after excluding all drugs without price decrease i.e., a total of 2562. The latter were drugs with no price change (either the price before and after the price revision on the BO remained the same or the medicine was not on the price revision list) or new medicines introduced to the Moroccan pharmaceutical market and medicines with increases in the price.

**Inclusion and exclusion criteria:** the medicines included are those marketed in pharmacies in Morocco and purchased directly by patients during the study period. The study excludes medicines sold in the public sector or in hospitals, as well as other pharmaceutical products sold in pharmacies, in this case medical devices, vaccines and dietary supplements and all medicines without price decreases over the study period. The term “medicine/drug” refers to a therapeutic presentation and not a brand name.

**Data source and variables:** an Excel database was developed exclusively for the 1704 medicines. The generic/originator status and the Anatomical/Therapeutic/Chemical (ATC) classification 1st level were added for each medicine. Ranges of PSP for medicines sold in pharmacies were created to assess the distribution of the drop according to the price range to which the drug belongs. To measure the distribution of the decrease across all the medicines concerned, the price differential was calculated firstly to obtain the value of the decrease in USD for each medicine and then to be able to calculate the percentage differential, i.e. how much its price has fallen as a percentage of its PSP. For international intelligibility, the price in “Moroccan Dirham (MD)” was converted into “USD”, with an average of 9.4384 MD for USD 1 according to international currency market data (2014-2019) and USD 1 was rounded to 10 MD.

**Statistical analysis:** the statistical analysis was performed using the Statistical Package for Social Sciences (SPSS) V26.0. For the calculation formulas adopted, the absolute price differential in USD was the difference between the price of medicine after it appeared in the OB and its price before revision. The relative price differential in percentage (%) was the ratio of the difference between the price of medicine after its appearance in the OB and its price before revision (numerator) to the price before revision (denominator). Results were presented in tabular form, both in numbers and in percentages.

## Results

**General characteristics:** the analysis showed that the price decrease affected 1704 medicines that were sold in Moroccan pharmacies during the study period, and the majority of those drugs were originator (54%). Anti-infective and cardiology medicines were the most affected, for a total of 40%, and alimentary tract and metabolism drugs represented 15.8%, while parasitological treatments constituted 0.5% (Table 1).

**Table 1:** characteristics of medicines affected by the price decreases (N=1704)

Medicine characteristics		N	%
Generic/originator status			
Generic		783	46
Originator		921	54
ATC classification 1^st^ level			
A	Alimentary tract and metabolism	221	13
B	Blood and blood-forming organs	65	3.8
C	Cardiovascular system	315	18.5
D	Dermatologicals	77	4.5
G	Genitourinary system and sex hormones	96	5.6
H	Systemic hormonal preparations, excluding sex hormones and insulin	26	1.5
J	Antiinfective for systemic use	386	22.7
L	Antineoplastic and immunomodulating Agents	57	3.3
M	Musculoskeletal system	100	5.9
N	Nervous system	194	11.4
P	Antiparasitic products, insecticides and repellents	8	0.5
R	Respiratory system	116	6.8
S	Sensory organs	29	1.7
V	Various	14	0.8

ATC: anatomical therapeutic chemical classification system

**Sales price ranges:** the price range 5< PSP≥ USD 10 was the most affected by a decrease (25.4%), followed by 10 < PSP≥ USD 50 (18.6%). The cheapest medicines with a PSP less than or equal to USD 1 and the most expensive with a PSP over USD 500 are the least affected. Of the 783 generics, the majority (48.1%) had a PSP between USD 5 and USD 20. No generic had a price above USD 500. The 921 originator medicines were found in all price ranges, with the majority (60.4%) having a PSP between USD 5 and USD 50 (Table 2).

**Table 2:** distribution of medicines according to the price range in USD (N = 1704)

Price ranges	All medicines		Originator		Generic	
	N	%	N	%	N	%
PSP≤ USD 1	10	0.6	5	0.5	5	0.6
1<PSP≤ USD 2	127	7.4	57	6.2	70	9
2<PSP≤ USD 3	138	8.1	66	7.2	72	9.2
3<PSP≤ USD 4	145	8.5	57	6.2	88	11.2
4<PSP≤ USD 5	119	7	57	6.2	62	7.9
5<PSP≤ USD 10	432	25.4	197	21.4	235	30
10<PSP≤ USD 20	317	18.6	175	19	142	18.1
20<PSP≤ USD 50	266	15.6	184	20	82	10.5
50<PSP≤ USD 100	89	5.2	72	7.8	17	2.2
100<PSP≤ USD 500	48	2.8	38	4.1	10	1.3
USD 500 and more	13	0.8	13	1.4	0	0
Total	1704	100	921	100	783	100PSP≤USD

PSP: Public Selling Price; USD: United States dollar

**Absolute price differential:** out of 1704 medicines, 22.8% saw decreases less than or equal to USD 0.1, and those of USD 50 or more were registered in 1.8% (31 medicines) with a maximum reduction of USD 743. For the 783 generics, 27.7% had 0.5 < Decrease≥ USD 2, and 25.7% had price drops less than or equal to USD 0.1, while the largest price reductions of USD 50 or more affected 0.3%. For the 921 originator drugs, 20.4% had a reduction less than or equal to USD 0.1 while 3.1% with drop exceeded USD 50 (Table 3).

**Table 3:** distribution of medicines according to the decrease in USD (N = 1704)

Decrease (USD)	All medicines		Originator		Generic	
	N	%	N	%	N	%
Decrease ≤ USD 0.1	389	22.8	188	20.4	201	25.7
0.1 < Decrease ≤ USD 0.5	331	19.4	157	17	174	22.2
0.5 < Decrease ≤ USD 2	364	21.4	147	16	217	27.7
2 < Decrease ≤ USD 5	250	14.7	152	16.5	98	12.5
5 < Decrease ≤ USD 10	170	10	113	12.3	57	7.3
10 < Decrease ≤ USD 50	169	9.9	135	14.7	34	4.3
USD 50 and more	31	1.8	29	3.1	2	0.3
Total	1704	100	921	100	783	100

USD: United States dollar

**Relative price differential:** for the distribution of the decrease on all the medicines, 28.7% has a decrease less than or equal 1% of the PSP and 4.6% reduced by more than 50% of which 0.3% affected more than 70% with a maximum of 73.21%. For generics, 28.4% were affected by a decrease less than or equal 1% and 0.6% of medicines saw the sharpest decreases, exceeding 70%. Originator drugs were found in all price drop ranges, with 29% decrease less than or equal 1% and 12.5% by 5% < Decrease≥ 10% (Table 4).

**Table 4:** distribution of medicines according percentage of decrease (N = 1704)

Decrease (%)	All medicines		Originator		Generic	
	N	%	N	%	N	%
Decrease ≤1%	489	28.7	267	29	222	28.4
1%< Decrease ≤ 5%	106	6.2	46	5	60	7.7
5%< Decrease ≤ 10%	291	17.1	115	12.5	176	22.5
10%< Decrease ≤ 15%	152	8.9	73	7.9	79	10.1
15%< Decrease ≤ 20%	102	6	53	5.8	49	6.2
20%< Decrease ≤ 25%	108	6.3	67	7.3	41	5.2
25%< Decrease ≤ 30%	114	6.7	63	6.8	51	6.5
30%< Decrease ≤ 40%	141	8.3	102	11.1	39	5
40%< Decrease ≤ 50%	122	7.2	86	9.3	36	4.6
50%< Decrease ≤ 60%	57	3.3	35	3.8	22	2.8
60%< Decrease ≤ 70%	17	1	14	1.5	3	0.4
70% and more	5	0.3	0	0	5	0.6
**Total**	1704	100	921	100	783	100

## Discussion

This is the first study in Morocco that analyzed all medicines sold in pharmacies that appear on price revision OB lists and on Morocco sales databases for a period of five years (2014-2019) after the implementation of the decree n° 2-13-852. Price decreases concerned 1704 medicines in all therapeutic classes, and were more important for originator drugs. For 22.8% of medicines, the decreases were ≥ USD 0.1, and 28.7% recorded a decrease of ≥1% of the public selling price. The price range 5< PSP≥ USD 10 was the most affected. A study carried out in Morocco showed that out of a total of 7373 registered medicines, 2619 were affected by a decrease after the adoption of the new pricing system [18]. According to academic research on the 1^st^ price revision list in April 2014 for medicines sold in pharmacies in Morocco, 1428 medicines were affected by the reduction out of a total of 4916 [19]. For our study, out of 4622 medicines sold in pharmacies, the decrease concerned 1704 medicines. The Moroccan Direction of Medicines and Pharmacy of MH announced that 1578 medicines received price reductions in 2014, including treatments for cardiovascular diseases, metabolism, and diabetes, as well as antibiotics and antineoplastic [20]. These results are in line with those obtained in our study, which showed that cardiology, digestive system, and metabolism drugs, as well as anti-infectives, were the most affected by the decrease. In terms of percentage, the same Ministry announced that the decreases recorded ranged from 20% to 80% of the PSP [20].

A price analysis in 2014 revealed that of the 1948 medicines whose prices fell, the percentage drop ranged from 0.006% to 78.6% of the PSP [21] while another showed that 28.9% had reductions of less than 5%, 13.8% had reductions of between 20% and 30% of their PSP and 0.4% saw their price fall by more than 70% [19]. In our study, out of a total of 1704 medicines, five recorded reductions exceeding 70%, with a maximum of 73.2%, and the majority, i.e., 489 medicines (28.7%), recorded a reduction of ≥1% in the PSP. The maximum decrease in the initial price of the drug recorded was USD 743, while the majority of reductions recorded were less than USD 0.1 (22.8%), and reductions of USD 50 or more concerned 31 drugs (1.8%). According to data from the National Social Security Fund of Morocco, 1591 drugs have decreased in price, namely 21.5%, the majority of which (60%) were originators, 954 in number, and 637 generics [9]. These are similar to the results of our study, through which, for a total of 1704 drugs affected by the decline, the originators were 921, representing 54%, and recorded reductions not exceeding USD 0.1 for 20.4%. Of the 783 generics, a quarter saw decreases of less than or equal to USD 0.1, and 27.7% fell by USD 0.5 and USD 2, while two drugs saw their prices fall by more than USD 50. Internationally, a study on the impact of the change in policy on medicine prices in Egypt had concluded that the application of external referencing pricing introduced a price reduction for expensive originator drugs [22].

A study carried out in Algeria on the introduction of the reference price concluded that originators were the most affected by the price decrease, but did not have a significant impact on generic prices [23]. Another study showed that the use of the reference price for the pricing of medicines introduces price reductions for both generic and originators drugs, but the latter nevertheless record the largest decrease [24]. Financial access to treatment still weighs on households and the downward trend in prices alone cannot improve access to medicines, especially for chronic diseases that require permanent treatment. According to the Moroccan National Health Insurance Agency, 3.2% of the population suffers from at least one long-term conditions [25]. Despite the evolution of Moroccan consumption of the drug, it remains very low compared to other countries with an average of USD 248 per capita in European countries and USD 576 in the United States, while it does not exceed USD 43.1 in Morocco [9]. The policy of comparison with other countries for the purpose of setting prices is one of the elements influencing the continuous evolution of prices [26] especially since it is closely dependent on price changes in the Benchmark countries, some of which are among the top 10 pharmaceutical markets in the world, namely France and Spain [27]. However, price decreases may jeopardize access to medicines [28] and the market may no longer be attractive to manufacturers as a result of the policy of reductions, in addition to the problem of the unavailability and withdrawal of certain medicines from the market [29].

**Limitations:** this study has some limitations to note. Our research describes the evolution of the decrease prices of medicines sold in Moroccan pharmacies and does not cover the pharmaceutical market as a whole. We were confronted with the limited amount of research dealing with drugs in pharmacies specifically. Most studies described general changes also involving hospitals. The majority of international research consulted on price changes focused on both decrease and increase. Our study was limited to the variables studied, without including data on value and volume of sales.

## Conclusion

Price revisions of medicines in Morocco have introduced decreases, the largest of which have not exceeded USD 0.1 and 1% of the PSP, and have affected more than half of the originator medicines. The current project of extending medical cover to the entire population and exemption from VAT for all medicines since January 2024 could be a way of improving access. Promoting generic medicines through an effective and efficient strategy remains important, especially as the price of drugs remains a challenge for the country. Medicine consumption research following the price decrease will be interesting to evaluate the impact of these revisions on access to medicines, also to understand the evolutions of national pharmaceutical market.

### What is known about this topic

- Regulation of medicines prices in Morocco dates back to 1969 and several studies have demonstrated the high price of medicines in Morocco;
- In December 2013, Morocco adopted Decree No. 2-13-852 on new methods for fixing prices of medicines;
- Several lists of price revisions have been published in official bulletins since April 2014, following the implementation of Decree No. 2-13-852.

### What this study adds

- 28 drug price revision lists were published between 2014 and September 2019; they not only included medicines whose prices had been revised downwards;
- Decreases affected drugs in all price ranges; the expensive medicines whose public selling price exceeds USD 500 were 13 and all were originators;
- Decrease ≥ USD 0.1 affected 22.8% of medicines sold in pharmacies and percentages of decline ranged from minus 1% to 73.2% as the maximum rate.
